# Dynamic decoupling of biomass and wax ester biosynthesis in *Acinetobacter baylyi* by an autonomously regulated switch

**DOI:** 10.1016/j.mec.2018.e00078

**Published:** 2018-09-22

**Authors:** Suvi Santala, Elena Efimova, Ville Santala

**Affiliations:** Laboratory of Chemistry and Bioengineering, Tampere University of Technology, Korkeakoulunkatu 8, FI-33720, Tampere, Finland

**Keywords:** WE, wax esters, AceA, isocitrate lyase, Lipid biosynthesis, Wax esters, Acetate, Dynamic control, Decoupling, Autonomous circuit

## Abstract

For improving the microbial production of fuels and chemicals, gene knock-outs and overexpression are routinely applied to intensify the carbon flow from substrate to product. However, their possibilities in dynamic control of the flux between the biomass and product synthesis are limited, whereas dynamic metabolic switches can be used for optimizing the distribution of carbon and resources. The production of single cell oils is especially challenging, as the synthesis is strictly regulated, competes directly with biomass, and requires defined conditions, such as nitrogen limitation. Here, we engineered a metabolic switch for redirecting carbon flow from biomass to wax ester production in *Acinetobacter baylyi* ADP1 using acetate as a carbon source. Isocitrate lyase, an essential enzyme for growth on acetate, was expressed under an arabinose inducible promoter. The autonomous downregulation of the expression is based on the gradual oxidation of the arabinose inducer by a glucose dehydrogenase *gcd*. The depletion of the inducer, occurring simultaneously to acetate consumption, switches the cells from a biomass mode to a lipid synthesis mode, enabling the efficient channelling of carbon to wax esters in a simple batch culture. In the engineered strain, the yield and titer of wax esters were improved by 3.8 and 3.1 folds, respectively, over the control strain. In addition, the engineered strain accumulated wax esters 19% of cell dry weight, being the highest reported among microbes. The study provides important insights into the dynamic engineering of the biomass-dependent synthesis pathways for the improved production of biocompounds from low-cost and sustainable substrates.

## Highlights

• Efficient conversion of acetate to storage lipids (wax ester) is demonstrated.
• AraC-pBAD promoter coupled with glucose dehydrogenase was used as a dynamic switch.
• The autonomous switch allowed dynamic shift from biomass to lipid synthesis mode.
• Wax ester yield and titer were improved by 3–4 folds over the wild type strain.
• The highest amount of wax esters produced in microbes, 19% of CDW, was achieved.

## 1 Introduction

Metabolic engineering and synthetic biology provide powerful means for the bio-based production of a variety of chemicals and other commodities by engineered microbes. Compared to conventional chemical synthesis, the superiority of biological production systems lies in the possibility to synthesize both the catalyst (i.e. the cell factory) and the product itself from very simple chemical compounds, such as sugars or organic acids. However, the challenge is to develop a system, which optimally distributes the resources and carbon flux between building up the catalyst and operating the catalyst for the actual production; extensive cell growth takes resources from the product synthesis, whereas too excessive flux towards product synthesis may result in reduced growth, insufficient cofactor regeneration, low enzyme expression levels, and eventually poor titers. To address the challenges related to the optimal distribution of cellular resources, a number of dynamic circuit designs targeting the central pathway nodes have been recently developed (Tan and Prather, 2017). While growth-associated genes responsible for central carbon metabolism cannot be directly deleted, various strategies for decoupling growth and product synthesis have been introduced; Soma et al. constructed a metabolic toggle switch in *Escherichia coli* for conditional knockout of citrate synthase *gltA,* an enzyme required for functional tricarboxylic acid (TCA) cycle (Soma et al., 2014). The switch allowed an induced shift of carbon flow from the TCA cycle to a synthetic isopropanol pathway. More recently, the system was further improved by introducing a sensor-regulator system responsive to a defined cell density (Soma and Hanai, 2015). Solomon et al. introduced a dynamic approach to controlling the glycolytic flux; antisense RNA technology and an inverting gene circuit were employed for inhibiting the activity of glucokinase (Glk), resulting in a controlled growth rate and a reduced production of acetate in *E. coli* (Solomon et al., 2012). Brockman and Prather introduced another example of a dynamic regulation system, where they developed a circuit for dynamic knockdown of phosphofructokinase-1 (Pfk-1), the enzyme responsible for the key step in the glycolytic pathway regulating glucose-6-phosphate flux (Brockman and Prather, 2015). By the temporal control of Pfk-1 degradation, glucose-6-phosphate could be efficiently directed to a heterologous *myo*-inositol synthesis pathway in *E. coli* instead of biomass production. In a more recent work, Doong et al. further improved the system with a *myo*-inositol responsive dynamic sensor that regulated the downstream enzymes of the pathway in converting *myo*-inositol to glucarate (Doong et al., 2018). The introduced systems represent elegant examples of advanced metabolic control, but complex circuit designs can be prone to destabilization in prolonged cultivations and function unexpectedly in scaled-up processes (Moser et al., 2012).

Microbial storage compounds, such as triacylglycerols (TAG) and wax esters (WE), are industrially relevant and desirable molecules due to their vegetable oil like properties and broad applicability in e.g. fuel, nutritional, cosmetic, and pharmaceutical industries. However, the synthesis of long carbon chain products derived from fatty acyl-CoA require significant energy investments from the cell. In addition, the synthesis of storage lipids directly competes with biomass production and is strictly regulated, growth-phase dependent, and requires high amounts of cofactors and excess carbon along with limitation on other nutrients, such as nitrogen (Wältermann and Steinbüchel, 2005). Therefore, it is difficult to simultaneously improve both the titer and the yield of such products. TAGs and WEs require the same key precursor, acyl-CoA, and the mechanisms and challenges behind their improved production are similar. While strategies for dynamically regulated production of free fatty acids (FFA) and FA derived products have been introduced (Xu et al., 2014, Teixeira et al., 2017), means for overcoming the challenges of storage lipid synthesis regulation are still lacking. Therefore, the production is conventionally improved by non-specific means, such as bioprocess optimization or in conditions with a defined carbon-nitrogen ratio (Kurosawa et al., 2010). As an example of a more advanced approach, Xu et al. (2017) established a semi-continuous culture system with model-aided bioprocess optimization and cell recycling, which allowed efficient TAG production and high productivities even with dilute acetate feed in *Yarrowia lipolytica*. While also metabolic engineering strategies have been employed for improving TAG (Runguphan and Keasling, 2014, Plassmeier et al., 2016, Tai and Stephanopoulos, 2013, Santala et al., 2011) and WE synthesis (Lehtinen et al., 2018a, Santala et al., 2014, Wenning et al., 2017) in microbes, efficient overproduction of acetyl-CoA coupled with dynamic resource distribution between biomass and storage lipid synthesis remains a challenge.

When microbes are cultivated on non-glycolytic substrates, such as acetate, the cells have to rely on an alternative route of TCA cycle, namely glyoxylate cycle. This route bypasses of the two decarboxylation steps of TCA cycle that yield CO_2_, allowing the cells to reroute the carbon for the synthesis of cell components. The key enzyme in the glyoxylate cycle is isocitrate lyase (AceA), responsible for converting isocitrate to glyoxylate and succinate. It was previously demonstrated that a knock-out of the isocitrate lyase in *Pseudomonas putida* improved the production of PHAs when grown on gluconate, apparently for providing surplus acetyl-CoA for the PHA synthesis (Klinke et al., 2000). Some bacteria also exhibit an alternative pathway for glycolysis, such as the modified Entner-Doudoroff pathway of certain *Acinetobacter* strains (Young et al., 2005, Kannisto et al., 2014). An interesting feature of the glycolysis of *Acinetobacter* is the oxidation of glucose to gluconate prior to the transport to cells. In the absence of glucose, the first enzyme of the pathway, a pyrroloquinoline-quinone (PQQ) dependent glucose dehydrogenase encoded by *gcd*, unselectively oxidizes other sugar compounds present in the medium, such as pentoses, without the capability to utilize them as a carbon source (Kannisto et al., 2015, Carr et al., 2003, Barbe et al., 2004). Importantly, this feature does not interfere with the utilization of non-glycolytic carbon sources, and can be considered being ‘orthogonal’ to the substrate utilization. Thus, this feature could be exploited in the regulation of metabolic pathways in the cells.

*Acinetobacter baylyi* ADP1 is a model laboratory strain derived from a soil bacterium (Vaneechoutte et al., 2006, de Berardinis et al., 2009). The strain has increasingly gained interest due to its wide substrate range (Young et al., 2005), the genetic tractability and easily engineered genome (Barbe et al., 2004, de Berardinis et al., 2008), and the ability to produce storage lipids (Santala et al., 2011, Kannisto et al., 2017) and non-native products (Lehtinen et al., 2017, Lehtinen et al., 2018b). Here, we used the strain as a scaffold for the construction of a dynamic switch for autonomous shifting of cells from the biomass mode to the storage lipid synthesis mode. We built a circuit for a conditional elimination of the glyoxylate cycle, which is the essential bypass for the cells growing on acetate and the key control node in lipid biosynthesis pathway. We demonstrate the functionality of the switch by the improved (both yield and titer) production of wax esters using acetate as a carbon source.

## 2 Material and methods

*A. baylyi* DSM 24193 (DSMZ, Germany), referred as *A. baylyi* ADP1, was used in the study. The single gene knock-out strain of *A. baylyi* ADP1Δ*aceA*::*tdk*/*Kan*^*r*^ (ACIAD1084 deleted) was kindly provided by Veronique de Berardinis (Genoscope, France) (de Berardinis et al., 2008). The complementation of *aceA* in the knock-out strain was carried out by traditional PCR-restriction cloning: a previously described integrative cassette (Santala et al., 2011, Santala et al., 2011) was used as the scaffold for the construction of a gene cassette with the *aceA* gene under an arabinose inducible promoter AraC-pBAD to replace *poxB* (ACIAD3381) in the genome of ADP1. The resulting strain *A. baylyi* ADP1Δ*aceA*::*tdk*/*Kan*^*r*^ Δ*poxB*::*araC*-pBAD-*aceA*-*Cm*^*r*^ was designated as ADP1-ara-aceA (for details, please see Supporting information). Transformations of ADP1 were carried out as described previously (Santala et al., 2011) with 25 µg/ml chloramphenicol. *A. baylyi* ADP1Δ*poxB*::*Cm*^*r*^ was used as the reference strain designated as ADP1-ref.

The strains were cultivated in modified MA/9 minimal salts medium (Lehtinen et al., 2018a) at 25 °C and 300 rpm unless stated otherwise. The medium was supplemented with 25–100 mM Na-acetate or 250 mM glucose, 0.1–0.2% casein amino acids (w/v), and 0–1.0% L(+)arabinose when appropriate.

The preliminary test cultivations for *A. baylyi* ADP1 Δ*aceA*::*tdk/Kan*^*r*^ and the wild type strain were carried out in 250 ml shake flasks. Fifty ml of parallel cultivations supplemented with 250 mM glucose and 0.2% casein amino acids were cultured for 48 h in 20 °C and 300 rpm, after which substrate utilization and WE production were determined.

The cultivations for optimizing the arabinose concentration for ADP1-ara-aceA growth were carried out using Tecan Spark® (Tecan, Switzerland) microplate reader in 25 °C for 21 h with two replicate wells for each strain and arabinose concentration. The medium was supplemented with 25 mM Na-acetate and 0.1% casein amino acids, and arabinose concentrations 0%, 0.05%, 0.1%, 0.2%, 0.5% or 1.0% were used for the induction of *aceA*. Mediums without acetate supplementation was used as the control medium to determine the growth on casein amino acids. The strain ADP1-ref was used as the positive control, whereas the knock-out strains ADP1 Δ*aceA*::*tdk/Kan*^*r*^ and *A. baylyi* ADP1Δ*aceA*::*tdk*/*Kan*^*r*^ Δ*poxB*::C*m*^*r*^ were used as negative controls for growth on acetate. For semi-quantitative determination of the WE production of ADP1-ara-aceA, the cells were cultivated for 30 h in two parallel 5 ml cultures in the same medium except with 50 mM Na-acetate in 5 ml tubes, after which biomass and WE production were determined. Arabinose concentrations 0%, 0.1%, 0.2%, 0.5% or 1.0% were used for the induction of aceA expression.

For the quantitative determination of the WEs with NMR, the strain ADP1-ref and ADP1-ara-aceA were cultured in total 600 ml of MA/9 medium supplemented with 50 mM Na-acetate, 0.1% casein amino acids, and 0.5% (~30 mM) arabinose divided in 12 Erlenmeyer flasks. HPLC samples were taken every 1–5 h and two parallel 40 ml samples for WE analyses were taken at time-points 5, 7, 9 and 10 h for ADP1-ref and 12, 16, 20, 24, and 38 h for ADP1-ara-aceA.

Biomass production was determined by optical density (OD_600_) or gravimetrically (as cell dry weight, CDW). Glucose, acetate, and arabinose concentrations as well as possible end-metabolites were determined by HPLC, and the WE production was determined by TLC or NMR as described previously (Santala et al., 2011, Lehtinen et al., 2018a, Santala et al., 2011). For details, please see Supporting information.

## 3 Results

**Fig. 1:**
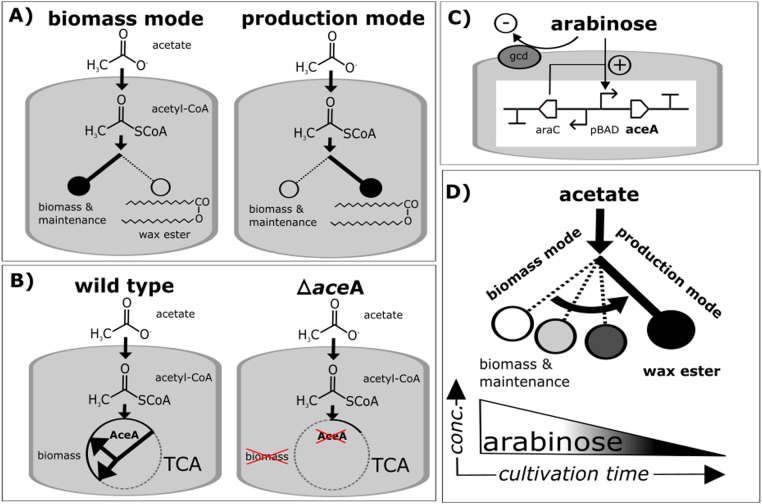
An autonomously regulated switch for shifting the cells from a biomass mode to a product synthesis mode. A) Wax ester (WE) synthesis strongly competes with biomass production; In the wild type cells, efficient WE synthesis is triggered only in defined conditions (i.e. in conditions with high carbon/nitrogen ratio). In normal growth conditions, most of the carbon is directed to biomass production and cell maintenance. B) The ADP1 wild type strain utilizes acetate for biomass production through a glyoxylate shunt in citric acid cycle. Isocitrate lyase (AceA) is the key enzyme in the glyoxylate shunt and thus essential for the growth on acetate. C) The genetic construct for dynamic regulation of aceA expression and growth. In the construct, aceA is placed under the arabinose-inducible promoter AraC-pBAD. When cultured on acetate, the arabinose used as the inducer is oxidized by the native glucose dehydrogenase (gcd) of ADP1, gradually reducing the expression of AceA. Arabinose oxidation does not serve as the carbon source or interfere with the acetate utilization, thus being orthogonal to the circuit function. D) Along with the inducer depletion (arabinose oxidation), the cells gradually shift from the biomass mode to the lipid synthesis mode; the less there is arabinose left in the culture, a higher proportion of the carbon flux is directed to the product. The amount of biomass can be simply regulated by adjusting the initial arabinose concentration.

In order to investigate the effect of the knock-out of isocitrate lyase on the growth and WE production in *A. baylyi* ADP1, we employed an *aceA* knock-out mutant strain of *A. baylyi* ADP1 (de Berardinis et al., 2008) for preliminary test cultivations. We observed that when grown on glucose, the cells grow more slowly, but produce WE titers comparable to those of the wild type (wt) strain; after 48 h of cultivation, wt had produced 470 ± 150 mg/l WEs compared to 460 ± 40 mg/l WEs produced by the knock-out strain. In opposite to the wt strain, however, the mutant strain did not exhibit growth on minimal medium supplied with acetate as the sole carbon source. This is due to the lack of a route for acetyl-CoA to be directed to biosynthetic pathways via malate. Thus, as acetyl-CoA represents the key precursor in both the biomass production through the glyoxylate shunt and the WE biosynthesis, we hypothesized that by dynamically regulating the isocitrate lyase, the state of the cells could be switched between biomass and lipid synthesis modes (Fig. 1). In order to make the shift dynamic, we introduced an approach for autonomous regulation of the isocitrate lyase AceA; by expressing the enzyme under an arabinose-inducible promoter AraC-pBAD, the induction is gradually repressed due to the depletion of arabinose by the glucose dehydrogenase activity of ADP1. The arabinose inducible promoter has been previously shown to function in *A. baylyi* ADP1 (Santala et al., 2014, Kannisto et al., 2014, Murin et al., 2012). In order to establish a system with maximal linearity and controllability, we constructed a gene cassette for genomic expression of *aceA* (Fig. 1c, d).

The gene cassette was introduced to the *aceA* knock-out mutant strain of ADP1 to replace a gene poxB (ACIAD3381), which has been previously shown to be a neutral target site in terms of growth and WE production (Santala et al., 2011, Santala et al., 2016). The resulting strain *A. baylyi* ADP1Δ*aceA*::*tdk*/*Kan*^*r*^ Δ*poxB*::*araC*-pBAD-*aceA*-*Cm*^*r*^ was designated as ADP1-ara-aceA, and the functionality of the complementation was experimentally confirmed (Fig S1). The strain *A. baylyi* ADP1Δ*poxB*::*Cm*^*r*^, designated as ADP1-ref, was used as the reference strain. Briefly, ADP1-ara-aceA did not grow or consume acetate in the absence of arabinose, indicating sufficiently tight regulation of the arabinose promoter, whereas with 1.0% arabinose the cells reached the same OD as ADP1-ref along with complete consumption of acetate. We also confirmed, that the AceA expression is reduced due to arabinose oxidation (Fig S2).

**Fig. 2:**
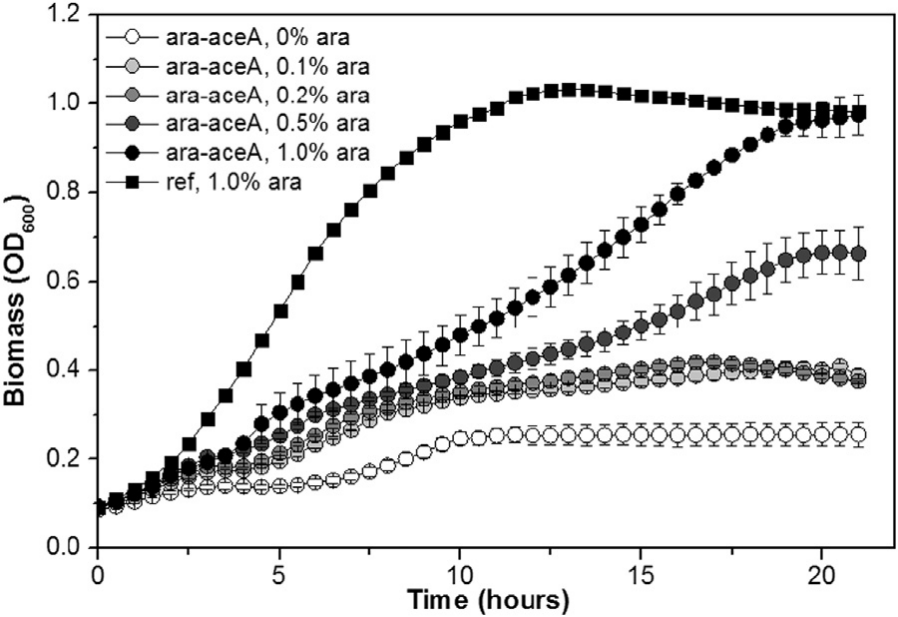
Growth of ADP1-ara-aceA and ADP1-ref with different arabinose concentrations. The cells were cultured in MA/9 medium supplemented with 25 mM acetate, 0.1% casein amino acids, and arabinose (0–1%) at 25 °C in micro well-plates for 21 h. The optical densities are presented as the average of three different replicate wells. For clarity, only the growth curve of the ADP1-ref culture containing 1% arabinose is shown. For ADP1-ref, the error bars are smaller than the symbols.

In order to find the optimal arabinose concentration in terms of both biomass and WE production, the strain ADP1-ara-aceA was cultivated in several different arabinose concentrations (Fig. 2). ADP1-ref was cultured as the reference strain. As indicated by the previous growth experiment, we found that 1% arabinose was sufficient to allow the engineered strain to reach the same biomass as ADP1-ref, albeit the cells grew slower. Within the concentration range 0–0.2%, only small differences in growth pattern or biomass production were observed. The slight increase in OD of uninduced cells is due to the utilization of the casein amino acids, which were added to the culture in order to promote the growth and to prevent nitrogen limitation; approximately the same amount of biomass is achieved without acetate supplementation with the wild type strain and the knock-out strain ADP1Δ*aceA::tdk/Kan*^*r*^Δ*poxB::Cm*^*r*^ with both acetate and casein amino acid supplementation (data not shown). For ADP1-ref, all the growth curves were similar regardless of the arabinose concentration used (data not shown).

**Fig. 3:**
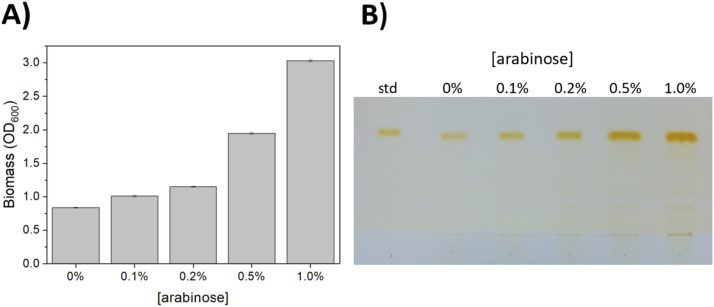
Biomass and WE production by ADP1-ara-aceA induced with different arabinose concentrations. ADP1-ara-aceA was cultured in MA/9 supplemented with 50 mM acetate, 0.1% casein amino acids, and arabinose (0%, 0.1%, 0.2%, 0.5%, or 1.0%) for 30 h. A) The amount of the produced biomass (at the end-point) was determined by optical density (600 nm) measurement, presented as an average of two individual replica cultures. B) The volumetric WE production in the cultures with different arabinose concentrations were determined by thin layer chromatography analysis. Jojoba oil was used as the standard.

Next, we determined which initial arabinose concentration most optimally distributes the carbon between the biomass and the WE production in the strain ADP1-ara-aceA. A clear correlation (R^2^ = 0.9968) between the arabinose concentration and biomass production (OD600) was detected (Fig. 3A). Without arabinose supplementation, the cells grew to an OD of approximately 0.8, which is due to the utilization of casein amino acids (the same OD was obtained with the knock-out strain ADP1Δ*aceA::tdk/Kan*^*r*^Δ*poxB::Cm*^*r*^). A semi-quantitative lipid analyses based on thin layer chromatography (TLC) was carried out to compare the amount of WEs produced (Fig. 3B). For all the cultures, the same sample volume was taken for the analysis, thus representing the titer of WEs produced. Based on image analysis, the intensity of the band representing the WE titer increases along with the biomass concentration. However, only a slight difference was observed between the bands of the 0.5% and 1.0% cultures, suggesting that in the culture which contains saturating amount of arabinose, a biomass respective to that of the wild-type strain is obtained, albeit the growth rate remains lower. When the intensities were divided with the optical densities, the highest amounts of WEs (per cell) were produced in the cultures with 0.2% and 0.5% arabinose. Thus, considering both the volumetric titer and the yield of WEs per biomass, the arabinose concentration of 0.5% was found to be optimal in terms of distributing the carbon and cellular resources between biomass and WEs.

**Fig. 4:**
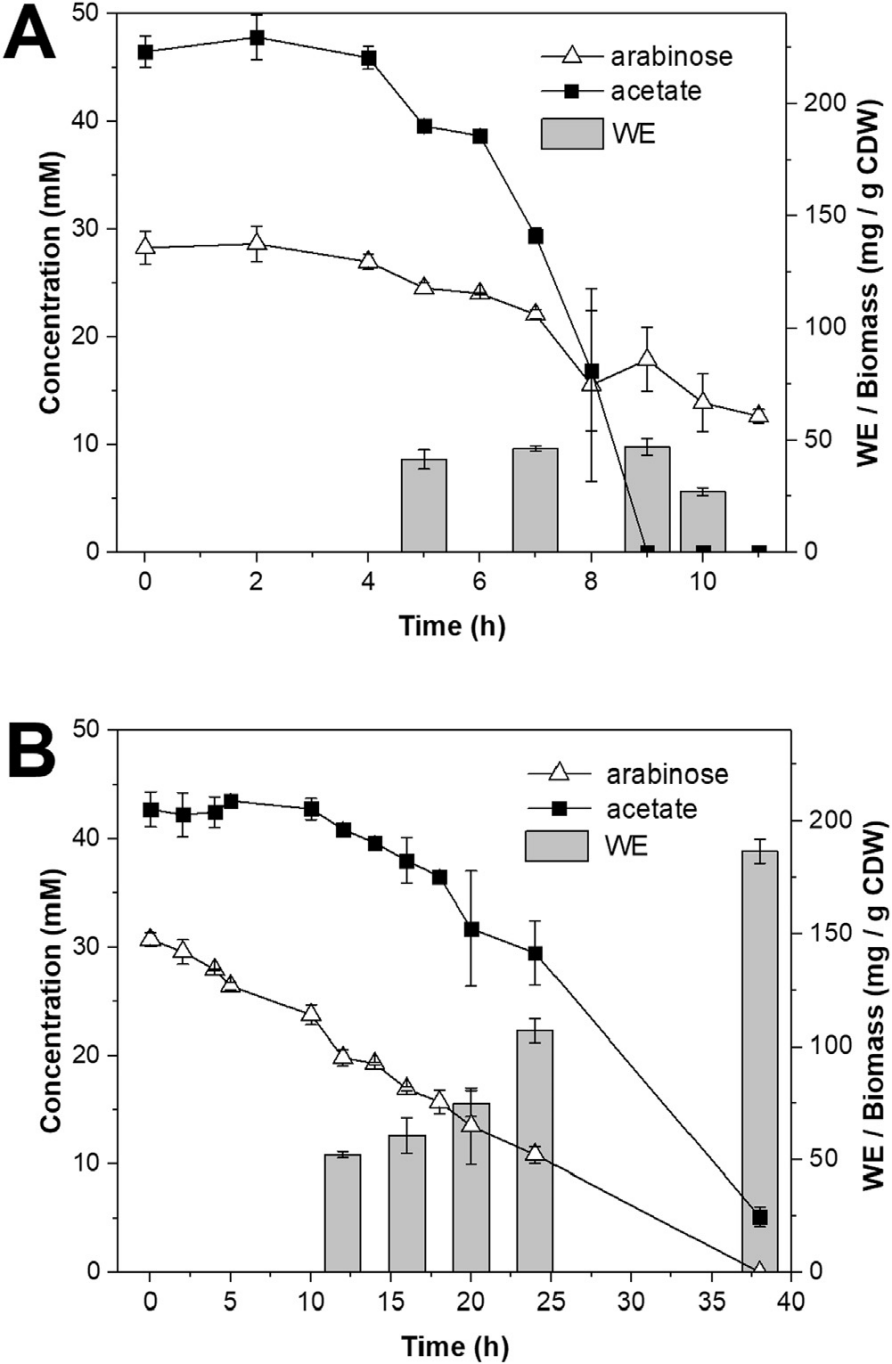
Acetate utilization, arabinose oxidation, and the WE accumulation in the batch cultures of A) ADP1-ref and B) ADP1-ara-aceA. The cells were cultivated in MA/9 medium supplemented with 50 mM acetate, 0.1% casein amino acids, and 0.5% arabinose for 12 h (ADP1-ref) or 38 h (ADP1-ara-aceA). For the arabinose and acetate concentrations determined by HPLC, the average and standard deviation for samples from two individual cultures are presented. Similarly, the WEs were quantitatively analyzed by NMR from two individual cultures. The WE production at different time-points is presented as mg/g CDW to demonstrate the different accumulation patterns of the strains.

Batch cultivations for ADP1-ref and ADP1-ara-aceA were carried out to determine the substrate and inducer consumption as well as to quantitatively determine the WE production (Fig. 4). ADP1-ref consumed all the acetate in 9–10 h; the amount of biomass increased until the 10-h time-point reaching CDW 1.3 g/l. The highest amount of WEs was measured at the 9-h time-point, being 47 mg/g CDW or 60 mg/l. In ADP1-ref, the WEs accounted for 44% of total lipids. The WE yield was found to be 0.02 g WE/g consumed acetate. The strain ADP1-ara-aceA utilized acetate more steadily and produced less biomass compared to ADP1-ref; the growth ceased after 16 h along with the arabinose depletion, and the biomass remained at 0.6–0.7 g/l CDW between the 16–24 h of cultivation. Thereafter, the WE content of the cells strongly increased, being highest at 38 h time-point, which also increased the amount of total biomass to 1.0 g/l. The WE titer was found to be 184 mg/l representing 19% of CDW, which was 3.8-fold higher compared to the ADP1-ref. In addition, the WEs accounted for 80% of all cellular lipids in ADP1-ara-aceA. The WE yield was 0.08 g WE/g consumed acetate, also being 4-fold higher over the ADP1-ref.

## 4 Discussion

Sugars, mainly glucose, have been the major carbon source for the heterotrophic microbial production of fatty acid derived compounds, such as TAGs and WEs, which can be used for the production of biofuels, biochemicals, and other biocommodities (Marella et al., 2018). However, in order to increase the feasibility and sustainability of the processes, utilizing alternative carbon sources is of high interest. Organic acids, such as acetate, serve as low-cost, abundant carbon sources for microbial lipid synthesis. Acetate can be readily derived from the hemicellulose fraction of plant biomass or waste streams, or produced from syngases by microbial fermentation (Bengelsdorf et al., 2013). For example, we have previously demonstrated the conversion of CO_2_ to WEs via acetate intermediate by combining microbial electrosynthesis with aerobic lipid synthesis in a two-stage process (Lehtinen et al., 2017). Many of the potential acetate streams, however, may have dilute acetate concentrations, thus sustaining conditions that are preferable for biomass production rather than for efficient storage carbon synthesis. On the other hand, highly concentrated acetate feeds can inhibit cell growth (Trček et al., 2015).

Nitrogen starvation is a commonly used and efficient means to trigger storage lipid accumulation in microbes (Breuer et al., 2012, Alvarez and Steinbüchel, 2002, Ishige et al., 2003). However, in such conditions the cell biomass typically remains low (Amara et al., 2016), which can result in lower overall product titers. Thus, genetic reprogramming would serve as a means to bypass the natural regulation for storage lipid synthesis. Mechanisms behind the regulation in microbes are not well understood, and therefore it has been challenging to genetically drive cells to overproduce the storage compounds or other fatty acid derived products. Previous strategies include for example the manipulation of the conserved carbon storage regulator CsrA through the CsrA-CsrB ribonucleoprotein complex, by which alterations in the central carbon metabolism and fatty acid synthesis regulation have led to favourable changes in both native and non-native product synthesis pathways in *E. coli* (McKee et al., 2012). This approach, however, is rather unspecific and potentially difficult to combine with other (targeted) engineering strategies.

In this study, we developed an autonomously regulated circuit for programmable synthesis of WEs in a native production host, *A. baylyi* ADP1. The circuit allows the cells to shift from the biomass mode to the WE synthesis mode independent from the carbon/nitrogen ratio or the growth phase of the culture. In practice, we replaced the native isocitrate lyase *aceA* with an arabinose inducible system, which allows a conditional and timed knockdown of the expression of *aceA*. This enzyme is essential for the biomass production when the cells grow on acetate. The timed reduction of *aceA* expression is achieved by gradually eliminating the inducer, namely arabinose; the native enzyme activity of the glucose dehydrogenase of *A. baylyi* oxidizes arabinose to arabino-lactone and further to arabonate, which in turn cannot serve as inducers. Importantly, and in contrast to other auto-induction-based systems, arabinose oxidation does not interfere with the utilization of the carbon source, here acetate, and can be thus considered as an orthogonal system. By adjusting the arabinose concentration, a predefined and optimal amount of biomass can be produced. When the inducer concentration is oxidized below the ‘threshold’ concentration, the cells shift from the biomass producing mode to the synthesis mode, efficiently directing carbon to product synthesis.

We confirmed that the engineered strain ADP1-ara-aceA with complemented isocitrate lyase was able to grow on acetate as the sole carbon source, when provided with arabinose. We observed that without the presence of arabinose, the cells did not exhibit growth, showing phenotype similar to the knockout strain *A. baylyi* ADP1Δ*aceA*::*tdk*/*Kan*^*r*^Δ*poxB*::*Cm*^*r*^. In addition, the obtained biomasses were in correlation with the initial arabinose concentrations between 0.2% and 1.0%; the arabinose concentration 1% was found to be saturating in terms of allowing ADP1-ara-aceA to reach at least the same biomass as the ADP1-ref, whereas the concentration 0.2% was found to be the ‘threshold’ for sufficient biomass production. The arabinose concentration 0.5%, in turn, allowed the cells to grow to a biomass approximately 50% of that of the ADP1-ref. Below 0.2% arabinose, the cells produced only slightly more biomass compared to the uninduced cells, indicating that arabinose concentrations> 0.2% are required for sufficient growth in the studied conditions. This finding was also supported by the reporter induction test; when the cells containing the bacterial luciferase *luxAB* under arabinose promoter were induced with the supernatant from different cultivation time-points (thus having different arabinose concentrations), clear induction of *luxAB* determined as luminescence production was only observed with the sample that contained> 0.2% arabinose.

According to the semi-quantitative WE analyses, the cultures supplemented with 0.2% or 0.5% arabinose produced the highest WE yields (per biomass). The cultures that were induced with 1% arabinose produced nearly two times more biomass compared to that of the 0.5% culture but had lower WE yield, indicating that a significant proportion of the carbon was directed to the biomass when 1% arabinose was used. Interestingly, the cultures with little (0.1%) or no arabinose produced the lowest WE yield, suggesting that at least subtle levels of *aceA* expression are required, not only for biomass production but also to support WE synthesis. The WE titers (WEs/volume) of 0.5% and 1.0% cultures were estimated to be very close to each other in the two cultures, whereas 0.2% culture had clearly lower volumetric WE production due to low biomass production. Thus, we considered 0.5% arabinose as the most effective inducer concentration in terms of optimal distribution of carbon between biomass and products.

For validation of the system, batch cultures for the ADP1-ref and ADP1-ara-aceA were carried out. It was shown that the engineered strain ADP1-ara-aceA efficiently accumulated WEs in simple batch conditions supplied with relatively low substrate concentration (50 mM acetate) and in non-optimal carbon-nitrogen ratio. The strain ADP1-ara-aceA produced 187 mg/l WEs representing 19% of the CDW and a yield of 0.08 g WE/g consumed acetate. As expected, the strain grew more slowly and produced less biomass than ADP1-ref, which resulted the overall productivity to be approximately 27% lower than in ADP1-ref. However, the yield of WEs per biomass and per consumed acetate were 3.8 and 4 folds higher compared to ADP1-ref, respectively. In addition, the WE titer was found to be 3.1 folds higher compared to that of the ADP1-ref. Thus, the dynamic regulation not only improved the yield of WEs per biomass and per used carbon, but clearly excelled the volumetric titer of that of the reference strain. For comparison, in a previous study (Lehtinen et al., 2017), *A. baylyi* ADP1 produced WEs from acetate with a titer of approximately 90 mg/l (from higher initial acetate concentration, 100 mM), with an average yield of 4% (carbon/carbon), being equal to 0.02 g WE/g consumed acetate. The highest WE titer reported so far has been 450 mg/l, which was obtained when the key enzyme of the pathway (fatty acyl-CoA reductase) was overexpressed and 5% glucose was used as the substrate (Lehtinen et al., 2018a). However, the yields (0.04 g WE/g glucose and 12.5% WEs of CDW) were lower compared to this study. With external long-chain alkane supplementation, up to 17% WEs of CDW has been obtained in *Acinetobacter* sp. M-1 (Ishige et al., 2002). Wax esters have been also produced in non-native hosts; for example, Wenning et al. (2017) demonstrated the production of very long chain WEs in *Saccharomyces cerevisiae*, but the obtained yields remained modest, up to 1.2% of CDW after 48 h cultivation.

Notably, the amount of WEs per cell was nearly constant in ADP1-ref at the analyzed time points, being 3.9–4.3% of the CDW. In ADP1-ara-aceA, by contrast, the amount of WEs per cell strongly increased along with the arabinose depletion; the percentage of WEs per cell increased from 5.2% to 19% between the sampling points. This indicates linear response to changing arabinose concentrations, but the exact mechanisms of the arabinose promoter in ADP1 are not known at single-cell level. Although the shift from biomass mode to lipid mode was rather gradual, the highest increase in the WE content (from 7.5% to 19%) was achieved after the arabinose concentration reached the ‘threshold’ 0.2% (equivalent to 15 mM arabinose). Thus, the arabinose concentration 0.2% seems to be the key turning point in the cellular state.

In the batch culture, initial concentrations 50 mM acetate and 0.5% arabinose were used. By the end of the culture, the arabinose was completely oxidized, and only a small amount (5 mM) of acetate remained unutilized. By adjusting the substrate and inducer concentrations, the system is potentially scalable to a wide range of substrate concentrations. In the current system, the expression of *gcd* was under native regulation. In some *Acinetobacter* species, the expression of *gcd* has been shown to be constitutive and independent of the culture conditions (van Schie et al., 1984, van Schie et al., 1989), but for *A. baylyi* ADP1 the mechanisms are not known. By modulating the expression of *gcd* the system could be potentially further fine-tuned and controlled. Moreover, coupling this system with other engineering strategies, such as introducing additional knock-outs (Santala et al., 2011) and/or overexpression of key enzymes of the pathway (Lehtinen et al., 2018a) could further improve the WE production.

Considering not only the efficient redirection of carbon to product, but also the downstream processing, the purity of the product is important. In ADP1-ara-aceA, WEs constituted 80% of total lipids, indicating high purity of the desired product. In ADP1-ref, only 44% of the total lipids were WEs.

The results from different experiments indicate that at least low levels of isocitrate lyase are required to maintain WE production from acetate, potentially due to the requirements for cells to generate e.g. NADPH for the synthesis. This hypothesis was also supported by a further observation in an additional experimental set-up where the WE content of the *aceA* knockout strain did not increase after a transfer from a glucose medium to an acetate medium (data not shown). While arabinose concentration< 0.2% is not sufficient to promote biomass production, it allows the cells to synthetize and maintain the required cofactor balance, and to efficiently produce WEs.

Despite that the highest (1%) arabinose concentration allowed ADP1-ara-aceA to reach the same biomass as ADP1-ref, the growth rate of ADP1-ara-aceA remained lower. While the linear correlation between the inducer concentration and obtained biomass was good, it is possible that the overall efficiency of pBAD is low in ADP1; Murin et al. Murin et al. (2012) studied the expression of *gusA* under the AraC-pBAD system in *A. baylyi* ADP1 both in a multi-copy plasmid and in a chromosomal single-copy gene cassette. They found out that while the plasmid expression resulted in significant induction compared to the uninduced cells (>100 folds), no induction was detected for the chromosomal expression. They speculated that the endogenous *A. baylyi* sigma factors might not bind the pBAD promoter very tightly. This may explain why the promoter activity is not very high even in ‘saturating’ inducer concentrations. In addition, it should be noted that the presence of acetate probably increases the activity of the isocitrate lyase (Hoyt et al., 1991), which further promotes the growth of ADP1-ref in the studied conditions. One might also speculate that higher growth rate of ADP1-ara-aceA would be counterproductive in terms of WE yield and titer; both substrate costs and downstream processing represent major factors in bioprocesses, further emphasizing the relevance of improved yield and product purity.

Rapid advancements in the CRISPR/Cas9 technologies have broaden the tools available for targeted genome engineering, and especially the employment of the deactivated Cas9 (dCas9) has recently gained interest in the context of targeted gene silencing (Larson et al., 2013). While the dCas9-based tools have been shown to be functional and applicable in a wide range of (microbial) hosts, challenges related to unpredictability, cellular burden and off-targeting may limit its use (Nielsen and Voigt, 2014, Cui et al., 2018). In addition, at least two different constructs are typically required for the fine-tuned expression of dCas9 and the RNA elements, and the system cannot be easily operated ‘hands-free’ without a timed addition of an inducer. In this context, the system described here provides a straight-forward, readily controllable, and reliable set-up for conditional and timed gene knock-down. In addition, other interesting synthetic biology hosts such as *P. putida* (Nikel et al., 2016) exhibit the same glucose dehydrogenase activity and could thus find this strategy applicable. However, the transferability of this system to others hosts such as *E. coli* and *S. cerevisiae* remains to be investigated in the future.

Our system serves as a simple and scalable method for dynamic, ‘hands-free’ regulation of growth-essential reactions in the cell, which allows targeted and adjusted biomass and product synthesis. Here, the dynamic regulation system was exploited in the conversion of acetate to carbon-rich storage compounds, namely WEs. The system allows efficient WE accumulation in a single process stage even in low substrate concentration. Without the dynamic regulation, excessive carbon source supplementation and fine-tuned nitrogen levels would be needed to induce the lipid accumulation, as well as separate process stages for biomass and product synthesis might be required. The introduced system could be potentially utilized and generalized for a broad range of synthesis pathways that are dependent on acetyl-CoA (Lehtinen et al., 2018a, Lehtinen et al., 2017, Lehtinen et al., 2018b). In addition, other sustainable carbon sources, such as lignin-derived compounds (Salmela et al., 2018, Salvachúa et al., 2015), or hemicellulose streams rich in acetate and arabinose, could be compatible with this system. However, the industrial relevance of this system remains to be evaluated in future along with further investigations.

## 5 Conclusions

We showed that an autonomously regulated genetic switch allowed the dynamic decoupling of biomass and WE production in engineered *A. baylyi* ADP1, which resulted in 3–4 fold improvements in the WE yield and titer compared to the reference strain. Shifting the cells from a biomass mode to a product synthesis mode was achieved by gradually repressing the growth essential gene *aceA* by a simple and robust set-up. The engineered strain produced 19% WEs of its cell dry weight, being the highest reported among microbes. The study demonstrates the possibility to bypass the challenges related to highly regulated storage lipid synthesis, and strengthens the status of *A. baylyi* ADP1 as a convenient host for metabolic engineering and high-value lipid production from sustainable substrates.

## Funding

This work was supported by the Academy of Finland (grant numbers 286450, 310135, 310188, and 311986).
