# Supplementary material for: Dynamic decoupling of biomass and wax ester biosynthesis in Acinetobacter baylyi by an autonomously regulated switch
Source: Metab Eng Commun. 2018 Sep 22;7:e00078. doi: 10.1016/j.mec.2018.e00078 (PMC6158957; doi:10.1016/j.mec.2018.e00078)
Supplement: Supplementary file 1 — Supplementary material [file mmc1.pdf]

Supplementary Information for

Dynamic decoupling of biomass and wax ester biosynthesis in *Acinetobacter baylyi* by an autonomously regulated switch

Suvi Santala, Elena Efimova, Ville Santala

Suvi Santala
Email: suvi.santala@tut.fi

Construction of genomic cassette for the complementation of AceA

For cloning and plasmid amplification, *Escherichia coli* XL1-Blue (Stratagene, USA) was used. The reagents and primers for molecular work were purchased from ThermoFisher Scientific (USA). The primers used in the study are presented in Table S1. The DNA fragment containing arabinose promoter pBAD and the constitutively expressed AraC regulator (1) designated as ara, were amplified from pBAV1C-ara-LuxCDE plasmid (2) using primers VS10_09 and VS10_10 and inserted to the plasmid i*luxAB_Cm*^{r}/pAK400c (3) using restriction sites MfeI and NdeI. The construction of the original integrative gene cassette is described more in detail in (4). The gene *aceA* (ACIAD1084) was amplified from the genome of ADP1 wt with primers SS17_08 and SS17_09 and cloned to the ara-i*luxAB_Cm*^{r}/pAK400c using restriction sites NdeI and XhoI to replace the genes *luxAB*. The resulting plasmid ara-i*aceA_Cm*^{r}/pAK400c was used for the transformation of of ADP1 as described previously (4); the gene cassette ara-i*aceA_Cm*^{r} integrates to the genome to replace the gene ACIAD3381 (*poxB*), resulting in strain *A. baylyi* ADP1Δ*aceA*::*tdk*/*Kan*^{r} Δ*poxB*::*araC*-pBAD-*aceA*-*Cm*^{r} designated as ADP1-ara-aceA. ACIAD3381 has been previously shown to be a neutral target site in terms of growth and WE production (3, 5). The wild type strain was transformed with the empty gene cassette i_*Cm*^{r}, resulting in the genotype *A. baylyi* ADP1Δ*poxB*::C*m*^{r}. This strain was designated as ADP1-ref, and used as the reference strain for ADP1-ara-aceA. The transformed cells were selected on LA plates containing 25 µg/ml chloramphenicol. All genetic modifications were confirmed with PCR and sequencing.

Verification of functional complementation of AceA

The functionality of the complementation in the strain ADP1-ara-aceA was confirmed in minimal medium MA/9 supplemented with 50 mM acetate as the sole carbon source (Fig S1). Arabinose (at concentrations 0, 0.1, and 1.0 %) was added to the cultures, and the cells were cultivated for 68 hours. Cells did not grow or consume acetate in the absence of arabinose, indicating sufficiently tight regulation of the arabinose promoter. Thus, without the arabinose supplementation, the cells showed phenotype similar to the knockout strain *A. baylyi* ADP1Δ*aceA*::*tdk*/*Kan*^{r}Δ*poxB*::C*m*^{r}. In the cultures with small amount of arabinose (0.1 %), the cells stopped growing after reaching an optical density (OD) of 0.3 and consumed only 5 mM acetate, whereas with 1.0 % arabinose the cells reached OD ~3.5 along with complete consumption of acetate. The ADP1-ref grew to slightly lower biomass (OD ~2.4) and consumed all the acetate; arabinose supplementation had no effect on the ADP1-ref growth. The slightly lower biomass of ADP1-ref might be due to the fact that the highest OD was not recorded during the cultivation, followed by decrease in the OD

due to the lack of carbon source. Thus, it was concluded that the arabinose concentration 1% is sufficient to allow the growth of ADP1-ara-aceA to reach at least the same biomass as the ADP1-ref. The control knockout strains ADP1Δ*aceA::tdk/Kan*^{r} and ADP1*ΔaceA::tdk/Kan*^{r} *ΔpoxB::Cm*^{r} did not exhibit growth nor acetate consumption with or without the presence of arabinose (OD 0 at 0-68 h).

The effect of arabinose oxidation on the induction of AraC-pBAD promoter. To further confirm that the AceA expression is repressed due to arabinose oxidation, i.e. the conversion of arabinose to non-inducive form, arabino-lactone and further to arabonate, we constructed a gene cassette similar to ara-aceA, except that the aceA gene was replaced with a bacterial luciferase genes *luxAB*. The gene cassette was transformed into the same genomic locus ACIAD3381 in the wild type ADP1. The strain ADP1-ara-aceA was cultivated in the MA/9 medium supplemented with 0.1 % casam, 50 mM Na-acetate and 0.5% arabinose, and supernatant samples were collected at time points 0, 12, 16, 24 and 38 h, representing remaining arabinose concentrations 0.46, 0.30, 0.26, 0.16 and 0 %, respectively, determined by HPLC. The samples were diluted (1:1) in fresh LB medium supplemented with 1 % glucose thus decreasing the final arabinose concentrations in half. The strain ADP1-ara-luxAB incubated with the supernatant-containing LB-medium for 6 hours in order to allow the induction of *luxAB* expression. Thereafter, 200 µl of cultures were transferred to 96 well plates as two replicates, and 5 µl of decanal stock (20 µl decanal per 1 ml 70% ethanol) was added in the wells. The bioluminescence signal was measured with Chameleon (Hidex, Finland) microplate reader and proportioned to the bioluminescence signal produced by the uninduced ADP1-ara-luxAB cells, referred as the background signal (Fig S2). We observed that the ADP1-ara-luxAB cultures that were incubated with samples that had more of its arabinose oxidized, produced lower luminescence signal, and with the samples in which the arabinose was completely oxidized, no luminescence signal above the background (uninduced) was detected. However, we also observed that with arabinose concentrations below 0.2% only low luminescence signals were obtained, indicating relatively low expression of *luxAB*.

Lipid and end-metabolite analyses

The amount of total lipids and WEs were estimated by TLC or quantified by NMR. For TLC, equal volumes of samples (3 ml) from different cultures were taken and the lipids were extracted using 'miniscale' chloroform-methanol extraction as described previously (6). Thirty µl of the chloroform phase was applied on 20 × 10 cm Silica Gel 60 F_{254} HPTLC glass plates with 2.5 × 10 cm concentrating zone (Merck, USA). Mobile phase used was n-hexane: diethyl ether: acetic acid 90: 15: 1 and iodine was used for visualization.Jojoba oil was used as the standard for WEs. For comparative evaluation of the intensities of the WE bands on TLC, the Gel analysis method of ImageJ software (rsb.info.nih.gov/ij/index.html) was applied as described in the ImageJ documentation.

For NMR analyses, the 40-ml biomass samples were freeze-dried and the cell dry weight (CDW) was determined gravimetrically. The lipid extraction and the quantitative ^{1}H NMR analysis of WEs was carried out as described earlier (3). The amount of total lipids was determined gravimetrically. The areas of the peaks in the NMR spectrum are directly

proportional to the molar concentration of each functional group, yielding specific concentration for WEs in total biomass. The concentration of WEs was calculated from the integrated signal at 4.05 ppm which is characteristic for protons of α-alkoxy-methylene group of esters (–CH2-COO-CH2–). For calculation of the WE titer in grams per liter, an average molar mass of 506 g/mol was used, based on GC analyses and the average chain length determined from NMR-spectra (6).

The glucose, acetate, and arabinose concentrations were determined by LC-20AC prominence liquid chromatograph (Shimadzu, USA) equipped with RID-10A refractive index detector, DGU-20A5 prominence degasser, CBM-20A prominence communications bus module, SIL-20AC prominence autosampler, and Shodex SUGAR SH1011 (Showa Denko KK, Japan) as described previously (4).

Table S1. List of primers used in the study.

Name	***Sequence (5'-> 3')***
VS10_9	CAATGAATTCCGATAAAAGCGGATTCCTGAC
VS10_10	ATCCCATATGTAATTCCTCCTGTTAG
SS17_08	AATACATATGACATATCAATCAGCTCTTGAGC
SS17_09	TAATACAATTGCGAACAGGCTTATGTCAAGACGTC

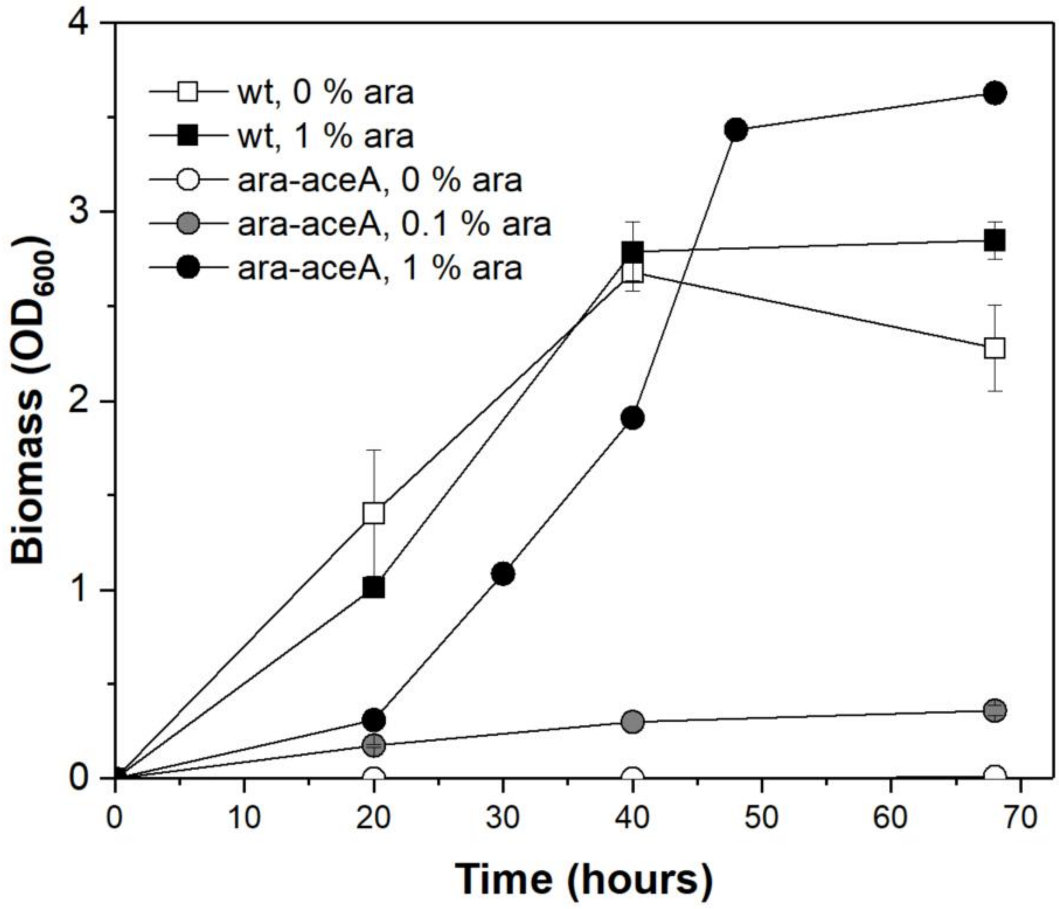

Fig S1. Growth of ADP1-ref and the engineered strain ADP1-ara-aceA in minimal medium. The cells were cultured for 68 hours at 25 °C in MA/9 medium supplemented with 50 mM Na-acetate as the sole carbon source. Arabinose concentrations of 0, 0.1, and 1.0% (for ADP1-ref 0 and 1%) were used for the induction. Optical densities representing biomasses are presented as an average of two individual replicates.

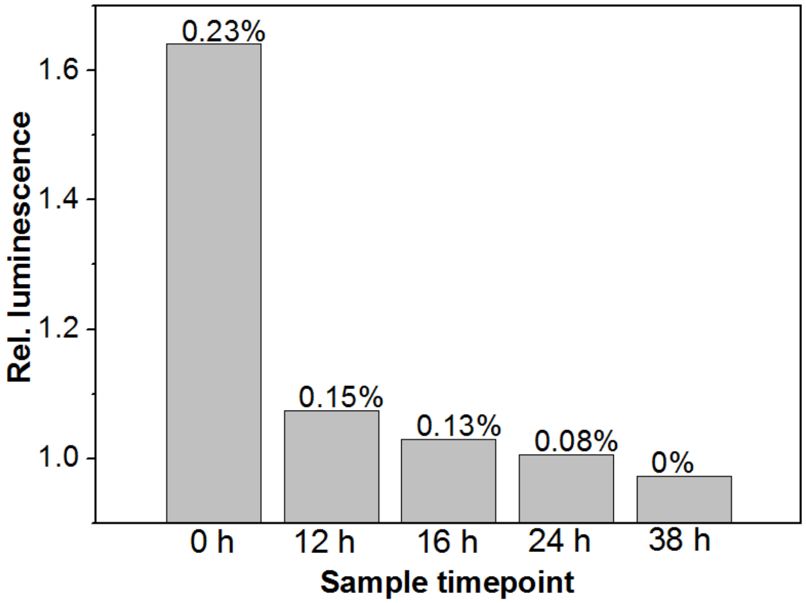

Fig. S2. Induction of ara-*luxAB* with samples having the arabinose partly oxidized. Cells expressing luxAB under arabinose-inducible promoter were incubated in LB medium with supernatant samples taken from ADP1-ara-aceA cultivation at different time-points. The expression of *luxAB* is observed as bioluminescence signal upon substrate (decanal) addition. In the figure, the percentages represent the amount of arabinose left in the ADP1-ara-aceA medium (diluted 1:1 in fresh medium) at the sampling time-point. The relative luminescence was calculated by dividing the bioluminescence signal with the signal detected from uninduced cells.

References

1. Guzman LM, Belin D, Carson MJ, & Beckwith J (1995) Tight regulation, modulation, and high-level expression by vectors containing the arabinose PBAD promoter. *Journal of bacteriology* 177(14):4121-4130.

2. Santala S, Efimova E, Koskinen P, Karp MT, & Santala V (2014) Rewiring the wax ester production pathway of *Acinetobacter baylyi* ADP1. *ACS Synth Biol* 3(3):145-151.

3. Santala S, Efimova E, Karp M, & Santala V (2011) Real-time monitoring of intracellular wax ester metabolism. *Microb Cell Fact* 10:75.

4. Santala S*, et al.* (2011) Improved triacylglycerol production in *Acinetobacter baylyi* ADP1 by metabolic engineering. *Microb Cell Fact* 10:36.

5. Santala V, Karp M, & Santala S (2016) Bioluminescence based system for rapid detection of natural transformation. *FEMS microbiology letters*.

6. Lehtinen T, Efimova E, Santala S, & Santala V (2018) Improved fatty aldehyde and wax ester production by overexpression of fatty acyl-CoA reductases. *Microbial Cell Factories* 17(1):19.
